# On Excision of Fistula in Ano

**Published:** 1892-03

**Authors:** A. S. Barling

**Affiliations:** House Surgeon to the North Staffordshire Infirmary


### Selections and Abstracts

### ON EXCISION OF FISTULA IN ANO.

BY A. S. BARLING, M. R. C. S.,
House Surgeon to the North Staffordshire Infirmary.

Fistula in ano is one of those diseases which do not tend to spontaneous cure. Certain slight cases doubtless get well if properly dressed, but in the large majority operative interference is necessary for their relief. The almost universal practice is to lay the sinus open, at the same time dividing the sphincter, and then to pack the gaping wound in such a way as to ensure its healing from the bottom. This mode of treatment is, I venture to submit, neither so simple nor yet so uniformly successful as we are generally given to understand. To carry it to the desired end it is almost essential that the patient should be kept in bed, the treatment not infrequently lasting for some months. I have traced several ordinary cases of fistula after their discharge from hospital, and in almost all the results have been disappointing. The average history seems to be somewhat as follows: The patient is operated on, and then dressed in hospital for a few weeks, during which time he makes rapid progress. He then begins to find the continuous rest irksome, and either on this account or because his bed is wanted, he becomes an out-patient. His friends are instructed, or even shown, how to dress the wound, and he goes out. He attends some time without making much improvement, and is then lost sight of. Some months elapse, when he again appears either very much in the same state as that in which he was last seen, or, worse still, with another fistula due to the bridging over of the wound. This is no fanciful picture, but one based on the careful observation of many cases. Such being the facts, the question naturally arises, can anything be done to shorten the time of cure without endangering its efficacy? Obviously the great drawback is that a large open wound is left to heal by granulation, and so must of necessity take a long time. Why should the wound be left open? Is it not possible, after removing all the granulation tissue, to sew the wound up with deep stitches, as is done in plastic operations for ruptured perineum. I have done this with very successful results, and will relate one recent case.

The patient was admitted into the North Staffordshire Infirmary, under the care of Mr. Alcock, suffering from a complete fistula, the external opening of which was 1 1-2 in. from the anal orifice, the internal about an inch up the rectum. I began the operation by thoroughly stretching the sphincter, dilating it first by means of a dilator, and then with fingers, until I could introduce my closed fist into the bowel, thus completely paralysing the muscle. I then slit up the fistula in the usual way. The granulating tract was cut and scraped away until two clean raw surfaces were obtained; these were then brought together over their whole extent by four silk stitches, extending deeply into the tissues below the wound, so that the bottom of the wound should be closed as well as the part nearer the skin. The silk had been boiled, and was put in by means of a curved Hagedorn's needle. Nothing untoward happened. The stitches were removed on the tenth day, when the wound was found to have healed soundly by first intention. Now, here is a man who was well in less than a fortnight after this operation, who, I believe under ordinary treatment would not have been cured for at least two months. Further, he had hardly any pain after the operation. The bowels did not move for a week, and the first motion brought about by repeated small doses of cascara, was not painful. The success of the proceedings is very largely due to attention to the following details: (1) Paralyse the sphincter; (2) clear away all granulations; (3) put the stitches in very carefully so as to secure accurate coaptation of the surfaces over their whole extent; (4) use aseptic silk so as to guard against the possibility of stitch abscess. Doubtless many surgeons have done this operation, and I shall be disappointed if I do not learn that it was the common mode of procedure with Hippocrates, but as I do not at present remember to have seen it described, I venture to call attention to it as worthy of notice. *The Lancet*, July 18, 1891, p. 123.—*Braithwaite's Retrospect.*

